# Retrospective assessment of the use of extended-release cabergoline in the management of equine pituitary pars intermedia dysfunction

**DOI:** 10.3389/fvets.2024.1332337

**Published:** 2024-03-06

**Authors:** Tania Sundra, Erin Kelty, Gabriele Rossi, David Rendle

**Affiliations:** ^1^Avon Ridge Equine Veterinary Services, Brigadoon, WA, Australia; ^2^School of Veterinary Medicine, Murdoch University, Murdoch, WA, Australia; ^3^School of Population and Global Health, University of Western Australia, Crawley, WA, Australia; ^4^EMT Consulting, Tiverton, United Kingdom

**Keywords:** horse, endocrine, laminitis, geriatric, dopamine, ACTH

## Abstract

**Introduction:**

Dopaminergic agonists are accepted as the most effective treatment for pituitary pars intermedia dysfunction. However, some horses are refractory to daily oral pergolide, the recommended registered treatment. Extended-release cabergoline (ERC) injection may offer an alternative. The objective of this retrospective case series was to describe clinical and endocrinological responses to ERC.

**Methods:**

Medical records of horses treated with weekly intramuscular injections of ERC (5 mg/mL, BOVA Aus) at either 0.01 mg/kg (high dose, HD) (*n* = 10) or 0.005 mg/kg (low dose, LD) (*n* = 30) were reviewed. Short-term ACTH responses were assessed at 5–8 days using a Wilcoxon signed ranked test. Longer-term ACTH responses (30 to 365 days) were assessed using generalised estimating equations.

**Results:**

Five to eight days after the first dose of LDERC, median adrenocorticotropic hormone (ACTH) concentration was lower (*p* = 0.001), changing from 153 pg/mL (IQR: 78, 331) to 57 pg/mL (IQR: 30, 102). With HDERC, median ACTH concentration was also 153 pg/mL (IQR: 96, 185) before and then 56 pg/mL (IQR: 29, 86) after 5–8 days of treatment (*p* = 0.047). Over 12 months of treatment, ACTH concentration ranged from 14 to >1,250 pg/mL (median: 51 pg/mL) in horses treated with LDERC and 20 to 472 pg/mL (median: 50 pg/mL) in horses treated with HDERC. Measurements remained above the seasonal reference range in 39.3 and 52.3% of horses treated with LDERC and HDERC, respectively. Clinical improvement was reported by owners in 78.3 and 100% of horses treated with LDERC and HDERC, respectively. Partial, self-limiting inappetence was reported in 30.0% of LDERC and 60% HDERC cases. Seven horses exhibited lethargy (5 LDERC, 2 HDERC). Insulin concentrations measured 30 days post-ERC treatment were no different from baseline.

**Discussion:**

Clinical and endocrinological responses were consistent with results of previous reports of oral pergolide treatment. Weekly injection of ERC may be an effective alternative to pergolide; the 0.005 mg/kg dose appeared to be as effective, with less risk of inappetence, than the 0.01 mg/kg dose that has been reported previously.

## Introduction

Pituitary pars intermedia dysfunction (PPID) is a neurodegenerative condition that results in loss of dopaminergic inhibition of the pars intermedia and leads to an overproduction of peptides, including adrenocorticotrophic hormone (ACTH) (1). PPID is the most common endocrine condition in geriatric horses and is encountered frequently in clinical practice (2). Epidemiological studies have demonstrated a disease prevalence of approximately 15–30% in horses over the age of 15 yrs (3). Clinical signs associated with PPID include hypertrichosis, laminitis, polyuria, polydipsia, lethargy, muscle wastage, and delayed wound healing (1, 4). In addition to good husbandry, the treatment of PPID involves the use of dopaminergic agonists, which reduce peptide secretion from the pars intermedia (1, 2, 5).

Pergolide, a dopaminergic agonist, has been used widely for the treatment of PPID for over two decades (5, 6). Pergolide is currently registered as tablet and (in some countries) liquid preparation; however, some horses are refractory to daily oral administration, and previous studies have reported that owners might be less committed to lifelong therapy in older animals (7–9). A recent study demonstrated poor compliance with the administration of pergolide tablets, and this may have implications for the control of clinical signs even though it did not appear to affect ACTH responses (9).

Cabergoline is a dopaminergic agonist that has the same mechanism of action as pergolide but is available as an extended-release intramuscular injection, alleviating the need for daily oral administration of medication (10, 11). For several decades, cabergoline was used to treat functional pituitary adenomas in humans, including cases refractory to pergolide therapy, as it has a high affinity for dopaminergic receptors in the hypothalamus and pituitary gland (12–16). Previous studies in horses have investigated the effects of cabergoline on prolactin, MSH and insulin concentrations (10, 11, 17) and shown that these hormones have reduced consistent with the drug having an inhibitory effect on both melanotrophs and lactotrophs in the equine pituitary gland.

Anecdotally, extended-release cabergoline (ERC) administered as a low-volume intramuscular injection is an effective alternative to pergolide. The objectives of the current study were to: (1) review and describe the initial (5–8 days) and longer-term (12 months) clinical and endocrinological responses to two dose rates of an intramuscular extended release cabergoline (ERC) injection that is being used widely in clinical practice for the treatment of PPID.

## Materials and methods

### Horses

Clinical records of privately-owned horses treated by Avon Ridge Equine Clinic (anonymised for peer review) between June 2021 and October 2022 were reviewed to identify horses that had been treated with ERC for the management of PPID. Horse weight was estimated using a validated weigh tape.^1^

### Treatment

In accordance with previous reports (10, 11, 17), horses initially received treatment with ERC as an intramuscular injection (5 mg/mL)^2^ at a “high” dose of 0.01 mg/kg (HDERC). Some horses were reported by owners to experience in appetence at this higher dose, and prompting a change in the practice protocol to use a “low” dose of 0.005 mg/kg (LDERC) for subsequent cases in an attempt to mitigate issues with appetite reduction.

Site selection and correct intramuscular injection technique were demonstrated by the treating veterinarian when administering the first dose. The injection site was cleaned with isopropyl alcohol^3^ prior to injection, and the total dose of cabergoline (maximum 1 mL) was administered at a single injection site in either the neck or gluteal musculature. Owners administered subsequent doses and were advised to record clinical changes and to contact their veterinarian immediately if any adverse effects were observed. Cabergoline is not currently registered for use in horses; thus, informed client consent for the use of an unregistered medicine was obtained prior to commencing treatment. Clinical signs were assessed and recorded at repeat veterinary examinations in consultation with the owners responsible for day-to-day care of the horses.

### Laboratory investigations

ACTH data was collated and grouped relative to cabergoline administration. For all cases ACTH results were available from up to 3 days prior to treatment, 5 to 8 days after the first injection of ERC, and thereafter at less consistent but regular intervals as dictated by the clinical progress of each case. Post-prandial insulin concentrations were also available from up to 3 days prior to, and after 30 (+/−3), days of treatment. Insulin was measured approximately 90 min after the provision of the horse’s normal feed and forage in the morning. Insulin and ACTH concentrations were measured using an Immulite 2000.^4^

### Data analysis

Signalment and clinical data were transposed from case records to Microsoft Excel^5^ for subsequent analysis. Age, sex, and baseline ACTH were compared between high and low doses of ERC using Wilcoxon sign-rank or Chi-squared tests. Median ACTH concentration and interquartile range (IQR) were calculated for baseline (up to 30 days prior to treatment), 5–8 days, 30–90 days, 91–180 days, and 181–365 days post treatment. Where horses had had ACTH concentration measured more than once during a period, the mean of the results was used. Each ACTH concentration measurement was also classified as being within or above the geographical and seasonal reference range (18) for the month of collection. The percentage of horses with one or more ACTH concentrations above the normal range was reported for each period.

Efficacy of HDERC and LDERC were examined by comparing the ACTH responses at different time points with baseline ACTH concentration. Comparisons between measures taken at baseline and 5–8 days were made using a Wilcoxon signed ranked test, overall and stratified by high and low dose. Comparison between ACTH concentration at each longer-term follow-up period and baseline was performed using generalised estimating equations, using a negative binomial distribution for ACTH values and a binomial distribution for normal/high ACTH values. Analysis was carried out in StataMP version 17.^6^

### Animal ethics approval

An ethics committee was consulted, and it was determined that ethics committee oversight was not required for the retrospective review of clinical data.

## Results

### Horses

Data from 40 horses were included. Mean (±standard deviation) age was 22.4 (±5.1 years), with 25 geldings (62.5%) and 15 mares (37.5%). The average weight of horses at baseline was 344 ± 146 kg. A variety of breeds were represented, including Welsh and Welsh crosses (*n* = 18, 45.0%), Thoroughbreds (*n* = 4, 10.0%), Standardbreds (*n* = 3, 7.5%), Quarter horses (*n* = 3, 7.5%), Shetlands (*n* = 2, 5.0%), Miniatures and Miniature crosses (*n* = 2, 5.0%), Warmblood and Warmblood crosses (*n* = 2, 5.0%), and a range of other mixed breeds (*n* = 6, 15.0%). There were insufficient data to compare groups but there were no obvious differences in breed distributions between the HDERC and LDERC groups. Horses started treatment at all times of year: January 9, February 2, March 0, April 1, May 3, June 0, July 7, August 7, September 1, October 2, November 2, and December 6, with no apparent difference between groups.

Clinical signs of PPID at initial evaluation included hypertrichosis (*n* = 28, 70.0%), weight loss (*n* = 10, 25.0%), laminitis (or a history of laminitis) (*n* = 10, 25.0%), muscle atrophy (*n* = 9, 22.5%), lethargy (*n* = 4, 10.0%), recurrent infections (*n* = 2, 5.3%), polyuria and polydipsia (*n* = 1, 2.6%) and poor wound healing (*n* = 2, 5.3%). Fifteen horses had previously received treatment with pergolide with a mean of 15.2 days (range 1–60 days) between receiving their last dose of pergolide and commencing treatment with ERC. Seven had received pergolide within a week of starting pergolide treatment (3 HDERC, 4 LDERC).

### Treatment

The most common reason for commencing ERC was actual or anticipated difficulty administering daily oral medication (*n* = 38, 97.4%). Three horses also displayed signs of partial anorexia whilst on treatment with pergolide at 2 mcg/kg. One horse demonstrated high ACTH concentration, complete anorexia and somnolence when treated with pergolide at 2 mcg/kg. Ten horses (25.0%) were treated with 0.01 mg/kg HDERC, 30 (75.0%) with 0.005 mg/kg LDERC. There was no significant difference between the two groups in terms of age (*p* = 0.253), sex (*p* = 0.187), or baseline ACTH (*p* = 0.179). Owners did not report missing any treatment doses.

At the end of the study period, 25 horses continued on treatment (5 HDERC, 20 LDERC) and seven horses were lost to follow up (3 HDERC, 4 LDERC). Of the horses that discontinued treatment, 2 (LDERC) stopped due to marked anorexia or colic, 2 due to owner finances (LDERC), 2 became refractory to injections (LDERC), 3 were euthanased for unrelated accidents or illness (2 LDERC, 1 HDERC), and 1 was switched to pergolide (HDERC).

### Short-term ACTH responses

Measurements of ACTH concentration were available for 32 horses on day 0 and after a single dose of cabergoline at days 5–8. The median ACTH concentration reduced from 153 pg/mL (IQR: 79, 245) to 57 pg/mL (30, 96) (*p* < 0.001). All horses had an ACTH concentration above the seasonal reference range prior to treatment; 19 horses (59.4%) remained above the seasonal reference after a single dose of cabergoline. Short-term ACTH responses stratified by dose are shown in Table 1; the reduction in ACTH concentration was significant in both groups.

**Table 1:** Short-term adrenocorticotropic hormone responses following a single dose of extended release cabergoline, stratified by low (LDERC) and high dose (HDERC).

	LDERC				HDERC			
	*n*	Pre-treatment^a^	Day 5–8	*p*-value	*n*	Pre-treatment^a^	Day 5–8	*p*-value
ACTH, median (IQR)	22	153 (78, 331)	57 (30, 102)	0.001	10	153 (96, 185)	56 (29. 86)	0.047
Above reference range^b^, *n* (%)	22	22 (100%)	13 (59.1%)	–	10	10 (100%)	6 (60.0%)	–

a 0 to 31 days prior to the commencement of treatment.

b Percentage of horses with one or more ACTH concentrations above the seasonally adjusted reference range.

### Longer-term ACTH responses

In horses with longer-term follow-up data available, ACTH concentration ranged from 59 to >1,250 pg/mL (median: 162 pg/mL) prior to treatment with all horses having an ACTH concentration above the seasonal reference range (Table 2). In the year following the commencement of treatment, ACTH concentrations ranged from 13 to 1,250 pg/mL (median: 50 pg/mL), with 46.4% of measurements remaining above the seasonal reference range. Longer-term ACTH responses are shown in Table 2 and are stratified by dose in Table 3. Reduction in ACTH concentration was significant with both doses at all time points, with the exception of the day 181–365 HDERC group.

**Table 2:** Adrenocorticotropic hormone concentration (pg/mL) in 40 horses treated with extended release cabergoline.

	*n*	Median (IQR)	*p*-value	Above reference^b^ (%)
Baseline^a^	40	162 (90, 286)	–	40 (100%)
Day 31–90	30	61 (41, 83)	<0.001	17 (56.7%)
Day 91–180	24	47 (38, 106)	<0.001	11 (45.8%)
Day 181–365	16	68 (50, 100)	<0.001	11 (68.8%)

a 0 to 31 days prior to the commencement of treatment.

b Horses with one or more ACTH concentrations above the seasonally adjusted ACTH range.

**Table 3:** Longer-term adrenocorticotropic concentration (pg/mL) following treatment with extended release cabergoline, stratified by low (LDERC) and high dose (HDERC).

	LDERC				HDERC			
	*N*	Median (IQR)	*p*-value	Above reference (%)	*N*	Median (IQR)	*p*-value	Above reference (%)
Baseline	26	170 (88, 331)	–	26 (100%)	9	150 (96, 178)	–	9 (100%)
Day 31–90	21	46 (32, 83)	<0.001	8 (38.1%)	9	67 (47, 80)	<0.001	9 (100%)
Day 91–180	16	48 (33, 119)	<0.001	8 (50.0%)	8	47 (42, 71)	<0.001	3 (37.5%)
Day 181–365	9	63 (34, 65)	<0.001	4 (55.6%)	7	102 (71, 280)	0.684	6 (85.7%)

### Insulin responses

Measurement of insulin concentration was performed at day 0 and day 30 in seven horses in the HDERC group. All horses were already being managed for equine metabolic syndrome with a diet consisting of <10% NSC for at least one week prior to measurement of insulin concentration. The diet of the horses remained consistent at both testing timepoints. Median insulin concentration prior to treatment was 185 μu/mL (IQR: 113, 279) and after treatment was 241 μu/mL (IQR: 113, 284) (*p* = 0.563).

### Longer-term clinical response to treatment

The owners’ perceived response to treatment was recorded in 31 of the 40 horses (23 LDERC and 8 HDERC). In the horses treated with LDERC, 78.3% reported an improvement (*n* = 17), while no change was reported by 13.0% of owners (*n* = 3) and 8.7% reported worsening of clinical signs (*n* = 2). Of the horses which improved, 77.8% demonstrated improved coat shedding (*n* = 14), 66.7% increased energy levels (*n* = 12), and 22.2% showed signs of improved body condition (*n* = 4). All owners reported good compliance with the weekly injection of ERC. Improvement was reported in 100% of the horses treated with HDERC (*n* = 8). Of these, 87.5% demonstrated improved coat shedding (*n* = 7), 25% increased energy levels (*n* = 2), and 25% showed signs of improved body condition (*n* = 2).

### Adverse reactions

Adverse events including self-limiting lethargy (HDERC: *n* = 2, 20%; LDERC: *n* = 5, 16.7%), partial anorexia (HDERC: *n* = 6, 60%; LDERC: *n* = 9, 30.0%), and mild colic (LDERC: *n* = 2, 6.7%) occurred within 12–36 h following the injection of ERC. Where partial anorexia was observed, horses displayed a preference for hay and grass over concentrate feed for approximately 12–24 h following injection with ERC. No reactions were reported at the injection sites in any of the horses that were treated.

The owners of one horse receiving HDERC opted to switch to pergolide after two injections, as anorexia and lethargy were observed for 12 h following each dose. The horse displayed no adverse effects when treated with pergolide. One horse receiving LDERC displayed lethargy and inappetence for 72 h. Treatment with LDERC was discontinued after the third dose, and the horse commenced treatment with pergolide at 2 mcg/kg. No adverse events were observed on pergolide. Of the three horses which demonstrated anorexia on pergolide, only one showed signs of partial anorexia (for 12 h) following treatment with LDERC. The horse which demonstrated marked anorexia and became an obtunded following each daily dose of pergolide displayed signs of partial anorexia for 18 h following each weekly injection with LDERC but remained clinically normal between doses after this time. Three horses that displayed partial anorexia on ERC (HDERC: *n* = 2; LDERC: *n* = 1) did not previously demonstrate any adverse effects on pergolide. One horse treated with LDERC was partially anorexic and displayed signs of low-grade colic for 6 days following the first dose. No further doses were administered, and no further episodes of colic or anorexia were reported.

## Discussion

In the horses studied, once weekly injection of cabergoline was associated with a reduction in ACTH concentration and an improvement in clinical signs of PPID at the previously reported 0.01 mg/kg dose and also at a lower 0.005 mg/kg dose.

Although median ACTH concentration decreased using both doses of ERC, ACTH concentration remained above the seasonal reference range in around half the treated horses. Whilst this appears disappointing, similar responses are identified in response to treatment of PPID with pergolide, with only 30% of horses having seasonally normal ACTH concentrations following treatment with this oral dopaminergic agonist (19, 20). Season has a profound effect on ACTH concentrations (21–23); however, it remains unclear whether the return of ACTH concentrations to within seasonal reference ranges should be an objective of treatment to optimise equine welfare in PPID (24). Inconsistent responses to treatment, as observed among horses treated with ERC in the present study, has also been reported following pergolide treatment (20). Inconsistency of response has been attributed both to inter-horse variability in pharmacokinetics and pharmacodynamics of dopaminergic agonists and to the heterogeneous and progressive nature of PPID (20, 25).

Whilst it can be challenging to assess objectively, clinical response to PPID treatment is ultimately more important than endocrine response (2, 24), and the owners of 83.9% of horses in this study reported improvement in clinical signs. However, owners were not blinded and were therefore subject to bias. Hypertrichosis, weight loss, history of laminitis, and muscle atrophy were the most common presenting signs in this study, consistent with previous reports (24, 26, 27). Decreases in hypertrichosis, laminitis occurrence and the reversal of weight loss and muscle atrophy have been used to assess clinical response to pergolide (24, 26, 27), with time to the improvement of clinical signs ranging from 2 months to 3–4 years (20, 26, 27). Clinical responses observed in this study are therefore consistent with those reported with pergolide. Treatment with ERC was not associated with post-prandial insulin responses in the small subset of horses where insulin measurements were performed. This is consistent with a previous study in horses which demonstrated that cabergoline does not affect the insulin response to a glucose challenge (17), and similar findings have also been reported with pergolide (28).

All but one owner in the current study cited difficulty in administering a daily oral medication as a reason to commence ERC treatment. All owners reported good compliance with ERC treatment. A study in Australia estimated that almost 70% of horses over 15 years of age lived exclusively at pasture, suggesting ageing horses are managed less intensively, which may make it more challenging and less appealing to medicate them daily in feed or by mouth (3). The extended-release nature of cabergoline offers an advantage in cases where daily administration of pergolide presents challenges with practicality and, therefore, compliance (29). Compliance in human and veterinary medicine is an emerging area of research in which equine medicine lags behind, with few studies having been performed. One report found that horse owners were less compliant compared to small animal owners, and veterinarians significantly overestimated client compliance (30). This is further supported by a recent survey from the UK, which compared the amount of pergolide used with the amount that should have been dispensed and showed that compliance was very poor, with only 48% of owners purchasing ≥90% of the amount required to supply the prescribed dose (9). This study also found that age and breed had a significant effect, with compliance being extremely low in owners of Shetland ponies and horses ≥26 yrs old (9). Previous studies have also demonstrated that routine health care was less frequently performed in aged animals (7, 8), suggesting that owners may be less committed to, and compliant with, healthcare recommendations in older horses (9). In addition to reducing the time and effort involved in treating PPID, the ERC injection allows for precise dosing, which may also offer advantages over pergolide tablets in smaller ponies where splitting of tablets may pose challenges for owners and potentially reduce compliance (9).

The HDERC initially used in this study was based on previous studies in horses (10, 11, 17). One report compared the effects of this 0.01 mg/kg dose of cabergoline (a different formulation to the one used in the current study) and pergolide on prolactin concentration and demonstrated that the suppressive effects of cabergoline lasted at least 10 days compared to an intra-muscular injection of pergolide, which only produced 24 h of suppression (11). Following injection every 10 days, cabergoline has also been shown to suppress plasma MSH concentrations (17). The authors’ anecdotal experience of using the ERC preparation used in the current study suggests a rapid onset of action and variable duration of effect, with ACTH concentration dropping within a few days and remaining suppressed for up to 2 weeks. In most horses, ACTH concentration appears to start to increase after 7 to 10 days hence the recommended 7 days treatment interval. Pharmacokinetic and pharmacodynamic studies of ERC in horses are required to determine the optimal dosing regimen. Preliminary observations from this study suggest the LDERC might be more appropriate for further study than the HDERC, as clinical responses were similar and there were less unwanted effects. Adverse events were noted in both groups; however, fewer cases of anorexia were reported in the LDERC compared with HDERC (30.0% vs. 60%), albeit case numbers were small. Anorexia is also reported following pergolide administration (1, 31); however, in this study, some horses demonstrated anorexia following pergolide administration but not ERC and vice versa. It is unclear why this is the case. Despite the variability which exists, ERC may offer an alternative treatment for horses which are unable to tolerate pergolide due to adverse effects.

Previous studies investigating the use of ERC in normal horses have not reported any adverse effects (10, 11, 17). In all cases where partial anorexia was reported in the current study, the owners observed that horses had a preference for long-stem forage (hay or grass) over cereal-based feeds. In humans, the use of dopaminergic agonists has been associated with feelings of nausea (32, 33), which may explain the reduction in feed intake in horses. A significant reduction in feed intake has also been observed in dairy cows following a single injection of ERC (34). The reduction in prolactin concentration that occurs with cabergoline administration (10) may suppress appetite, as prolactin has been shown to stimulate food intake in other species (34, 35). The two horses that suffered from anorexia for longer than 24 h had significant dental disease, both having lost several molars and having had incisors extracted for the treatment of equine odontoclastic tooth resorption and hypercementosis, a common disorder affecting older horses (36, 37). As a result, neither were able to chew long-stem forage. Whilst anorexia from pergolide administration might be resolved by abruptly reducing the dose (1), the long-acting nature of ERC does not allow for such rapid drug withdrawal. Until further investigations are performed, veterinary surgeons should be cognisant of the possibility that ERC may have a more profound effect on feed intake in horses with significant dental pathology.

This study provides preliminary data and is limited by retrospective data collection, lack of an untreated control or placebo group, small sample size, and short follow-up period. However, the results suggest that once weekly injection of extended release cabergoline may be an effective treatment for horses with PPID and provides a basis for designing more robust investigations. A dose of 0.005 mg/kg may be more appropriate for the treatment of PPID than the 0.01 mg/kg dose that has been reported in horses previously. Larger, blinded, randomised clinical trials and studies on the pharmacokinetics and pharmacodynamics of ERC are warranted.

## Data availability statement

The original contributions presented in the study are included in the article/supplementary material, further inquiries can be directed to the corresponding author.

## Ethics statement

The requirement of ethical approval was waived by University of Murdoch Animal Ethics Committee, Perth for the studies involving animals because collation of clinical data was retrospective. The studies were conducted in accordance with the local legislation and institutional requirements. Written informed consent was obtained from the owners for the participation of their animals in this study.

## Author contributions

TS: Conceptualization, Investigation, Methodology, Supervision, Writing – original draft, Writing – review & editing. EK: Conceptualization, Data curation, Formal analysis, Investigation, Methodology, Writing – original draft, Writing – review & editing. GR: Conceptualization, Formal analysis, Writing – review & editing. DR: Investigation, Supervision, Writing – review & editing.

## Footnotes

^1^Virbac, 361 Horsley Road, Milperra, NSW 2214, Australia.

^2^BOVA Aus, Suite 1, 304-318 Kingsway Caringbah, NSW 2229.

^3^Isopropyl Alcohol; PharmAust Manufacturing, Malaga, WA, Australia.

^4^Siemens, 885 Mountain Highway, Bayswater, Victoria 3,153, Australia.

^5^Microsoft corporation, Redmond, USA.

^6^StataCorp, Texas, USA.
